# Incidence of relapse following a new approach to simplifying and optimising acute malnutrition treatment in children aged 6–59 months: a prospective cohort in rural Northern Burkina Faso

**DOI:** 10.1017/jns.2021.18

**Published:** 2021-04-19

**Authors:** Maguy Daures, Kevin Phelan, Mariama Issoufou, Ousmane Sawadogo, Bruno Akpakpo, Moumouni Kinda, Susan Shepherd, Renaud Becquet

**Affiliations:** 1 University of Bordeaux, Inserm, French National Research Institute for Sustainable Development (IRD), Bordeaux Population Health Research Center, Team IDLIC, UMR 1219, Bordeaux, France; 2 The Alliance for International Medical Action (ALIMA), Paris, France; 3 The Alliance for International Medical Action (ALIMA), Yako, Burkina Faso; 4 Association KEOOGO, Ouagadougou, Burkina Faso; 5 The Alliance for International Medical Action (ALIMA), Dakar, Senegal

**Keywords:** Acute malnutrition, Children, Incidence, MUAC-based and reduced doses protocol, Relapse, West Africa, aHR, adjusted hazard ratio, AM, acute malnutrition, CHWs, community health workers, CIs, confidence intervals, CORTASAM, Council of Research & Technical Advice on Acute Malnutrition, HAZ, height-for-age *Z*-score, IQR, interquartile range, MAM, moderate acute malnutrition, MUAC, mid-upper arm circumference, OptiMA, Optimising treatment for acute MAlnutrition, RDT, rapid diagnostic tests, RUTF, ready-to-use therapeutic food, SAM, severe acute malnutrition, sd, standard deviation, WHO, World Health Organization, WHZ, weight-for-height *Z*-score

## Abstract

The present study aimed to determine the 3-month incidence of relapse and associated factors among children who recovered under the Optimising treatment for acute MAlnutrition (OptiMA) strategy, a MUAC-based protocol. A prospective cohort of children successfully treated for acute malnutrition was monitored between April 2017 and February 2018. Children were seen at home by community health workers (CHWs) every 2 weeks for 3 months. Relapse was defined as a child who had met OptiMA recovery criteria (MUAC ≥ 125 mm for two consecutive weeks) but subsequently had a MUAC < 125 mm at any home visit. Cumulative incidence and incidence rates per 100 child-months were estimated. Multivariable survival analysis was conducted using a shared frailty model with a random effect on health facilities to identify associated factors. Of the 640 children included, the overall 3-month cumulative incidence of relapse was 6⋅8 % (95 % CI 5⋅2, 8⋅8). Globally, the incidence rate of relapse was 2⋅5 (95 % CI 1⋅9, 3⋅3) per 100 child-months and 3⋅7 (95 % CI 1⋅9, 6⋅8) per 100 child-months among children admitted with a MUAC < 115 mm. Most (88⋅6 %) relapses were detected early when MUAC was between 120 and 124 mm. Relapse was positively associated with hospitalisation, with an adjusted hazard ratio (aHR) of 2⋅06 (95 % CI 1⋅01, 4⋅26) for children who had an inpatient stay at any point during treatment compared with children who did not. The incidence of relapse following recovery under OptiMA was relatively low in this context, but the lack of a standard relapse definition does not allow for comparison across settings Closer follow-up with caretakers whose children are admitted with MUAC < 115 mm or required hospitalisation during treatment should be considered in managing groups at high risk of relapse. Training caretakers to screen their children for relapse at home using MUAC could be more effective at detecting early relapse, and less costly, than home visits by CHWs.

## Introduction

Acute malnutrition is a major public health problem that affects 49⋅5 million children aged 6–59 months worldwide each year and contributes to nearly half of all annual childhood deaths^(1,2)^. One-fourth of this global burden is in Africa, and the largest number of children on the continent affected by acute malnutrition, 5⋅1 million, live in West Africa^(2)^. The World Health Organization (WHO) defines severe acute malnutrition (SAM) as mid-upper arm circumference (MUAC) <115 mm or weight-for-height *Z*-score (WHZ) < −3 or the presence of nutritional oedema, and moderate acute malnutrition (MAM) as MUAC between 115 and 124 mm or WHZ between −2 and −3 *Z*-score. Collectively, these categories are referred to as acute malnutrition (AM).

Current WHO guidelines for the management of SAM recommend that children who recover from an episode of SAM should be periodically monitored in order to detect relapse early^(3)^. Relapse after recovery has been published from several treatment programmes, but with widely varying criteria for anthropometry and time period of follow-up. In a MUAC-based programme, Binns *et al.* in Malawi found a relapse rate of 1⋅9 % at 3 months post-recovery among children admitted with MUAC ≤ 115 mm and discharged with a MUAC ≥ 125 mm for two consecutive weeks^(4)^. In Burkina Faso, Somasse *et al.* found a relapse rate (defined as WHZ < −2) of 15⋅4 % at 12 months post-recovery among children admitted with MUAC < 125 and discharged with a WHZ > −2^(5)^. Relapse under the reduced doses protocol has been also described once among SAM children in the Mango trial^(6)^. The study proposed a reduced dose from the third treatment week to discharge (1 sachet <7 kg, 2 sachets ≥7 kg) and described a relapse rate (WHZ < −3 or MUAC < 115 or oedema) over 12 weeks after recovery of 2⋅4 % in the reduced doses protocol *v.* 1⋅8 % in the standard protocol of Burkina Faso.

Factors associated with the risk of relapse are not yet clear and research focused mainly on socioeconomic, contextual and nutritional status. Having lower anthropometric measures at admission or discharge is the most common risk factor associated with relapse^(4,5,7,8)^. Recently, a case-control study in Ethiopia showed an increased risk of relapse among boys when compared with girls^(9)^. Socioeconomic risk factors like poor sanitary conditions and food insecurity were also associated with relapse^(5,9–11)^, but these findings were not consistent across studies^(10)^. Incomplete vaccinations or non-receipt of Vitamin A supplement were also associated with an increased risk of relapse^(5,9)^. A study in Malawi showed that children whose height-for-age *Z*-score (HAZ) declined in the year following recovery from MAM treatment had a higher risk of AM relapse^(12)^.

The Optimising treatment for acute MAlnutrition (OptiMA) strategy is a new approach to simplifying and optimising acute malnutrition treatment in children aged 6–59 months. The OptiMA strategy trains mothers to use MUAC bracelets for screening and targets treatment to children with MUAC < 125 mm or oedema with one product – ready-to-use therapeutic food (RUTF) – at a gradually reduced dose. Children with MUAC < 115 mm or oedema received 175–200 kcal/kg per d of RUTF. Children with MUAC 115–119 mm, either at admission or during the course of treatment, received 125 kcal/kg per d of RUTF, and children with MUAC 120–124 mm, either at admission or during the course of treatment, received 75 kcal/kg per d of RUTF. A single-arm proof-of-concept trial was conducted in 2017 in the 54 health facilities of Yako district in Burkina Faso (Passore Province, North Region) to evaluate this new strategy^(13)^. Children were considered eligible for enrolment in the OptiMA trial if they were aged 6–59 m with a MUAC < 125 mm or bipedal oedema and presented at any of the 54 health facilities in Yako District.

The WHO recommends that more research is needed in different contexts to improve our understanding of relapse rates, including potential risk factors, and to standardise child follow-up after recovery^(3)^. The present study aimed to determine the incidence of relapse, among children who recovered from a reduced doses protocol, the OptiMA strategy. We also propose to analyse the risk factors of relapse as anthropometric characteristics (MUAC at admission, weight gain and length of stay), child sociodemographic data, mother characteristics and hospitalisation in order to identify group at a high risk of relapse and prioritise the follow-up after treatment.

## Population and methods

### Study design

Within the OptiMA trial, a prospective cohort study was conducted at a randomly selected sample of health centres among all children who recovered from treatment, as defined in the following paragraphs.

A one-stage cluster sampling design was used and health centres were stratified by population size. Six health centres were excluded due to inaccessibility, and finally, twelve of forty-eight health centres were selected for the relapse study.

All children who were admitted in the OptiMA trial in one of the selected health centres, and achieved recovery defined as a MUAC ≥ 125 mm and no oedema for two consecutive weeks, a good clinical health and with a minimum programme stay of 4 weeks were included in the relapse study. The inclusions in the relapse study occurred from April to November 2017, and the follow-up of study participants ended in February 2018.

The expected number of children needed for the present study was 700, based on the assumptions of a relapse rate at 3 months of 15 %^(5)^ and a loss to follow-up rate of 10 % with a statistical precision of 4 % and a design effect of 2.

### Data collection procedures

Children were seen at home every 2 weeks for 3 months (six visits) by a community health worker (CHW). At each visit, the following data were collected: MUAC measured to a precision of ±1 mm, date of the visit, child's status (alive, absent and deceased), and whether the child had been readmitted for AM treatment since the last visit. Data on child sociodemographic and mother characteristics were collected during the first episode of acute malnutrition at the admission under the OptiMA protocol.

Data were manually recorded on an individual standardised form by the CHW supervised by a technical research assistant and then entered into an anonymized Access database. At the conclusion of the study, the last home visit was made to determine the vital status of children who had incomplete MUAC data.

### Relapse definition

Relapse was defined as a child who recovered under the OptiMA protocol and who withdrew from the anthropometric criteria at admission as MUAC < 125 mm or oedema during any of the six home visits planned for the present study.

When a relapse was identified, the caretaker was informed by the CHW of the child's nutritional status and asked to visit the health centre as soon as possible.

### Statistical analysis

Continuous variables were described in terms of mean (standard deviation, sd) and compared between included and excluded children, using independent sample *t*-test or Mann–Whitney test, according to the expected application conditions. Categorical variables were described in terms of frequency and compared between included and excluded children, using the *χ*^2^ test or exact Fisher test.

Following the Council of Research & Technical Advice on Acute Malnutrition (CORTASAM) guidance published in 2020, the prevalence, cumulative incidences and incidence rate (per 100 child-months) of relapse with 95 % confidence intervals (CIs) were calculated^(14)^.

The prevalence was defined as the number of children who relapse at 3 months after their discharge out of the total number of children seen during this sixth last visit. The cumulative incidence was defined as the number of children who relapse once from 0 to 3 months after their discharge as recovered out of the total number of children followed at least once in the study. The incidence rate was the total number of episodes of relapse per person-time rate (per 100 child-months). Monthly person-time was calculated as having been seen at least once in the first 31 d or/and 32–63 d or/and 64–100 d following discharge. A child has seen at least once during these three periods counts as three person-time. If a child has not been seen during one period, he was not counted as a person-time for this period. To describe the incidence rates according to the children nutritional status at admission, the incidence rates were also stratified by the MUAC categories during the first admission in the OptiMA programme and the MUAC categories at relapse.

Survival analysis to identify variables predictive of the 3-month relapse incidence was run using a shared frailty model, an extension of the Cox proportional hazard model^(15)^, with a random effect on health facilities, and by assuming a Weibull distribution for the baseline hazard function. A univariable analysis by fitting a separate model for each covariate was performed, and variables having a *P*-value ≤ 0⋅2 were entered into the multivariable analysis. A *P*-value < 0⋅05 was considered statistically significant in the final model.

All statistical analyses were performed with RStudio (RStudio, Inc., Boston, MA, USA).

### Ethics

The present study was granted ethical approval by the Ethics Committee for Health Research (2016-6-067) and the Technical Review Committee for Clinical Trial Authorizations (5003720165EC0000) of the Ministry of Health of Burkina Faso. The trial was registered on clinicaltrials.gov (NCT03027505). Caregivers gave written consent (signature or fingerprint) prior to enrolment for all children included in the study. All data was anonymized when entered into the database and unique identification numbers were coded.

## Results

**Fig. 1.**
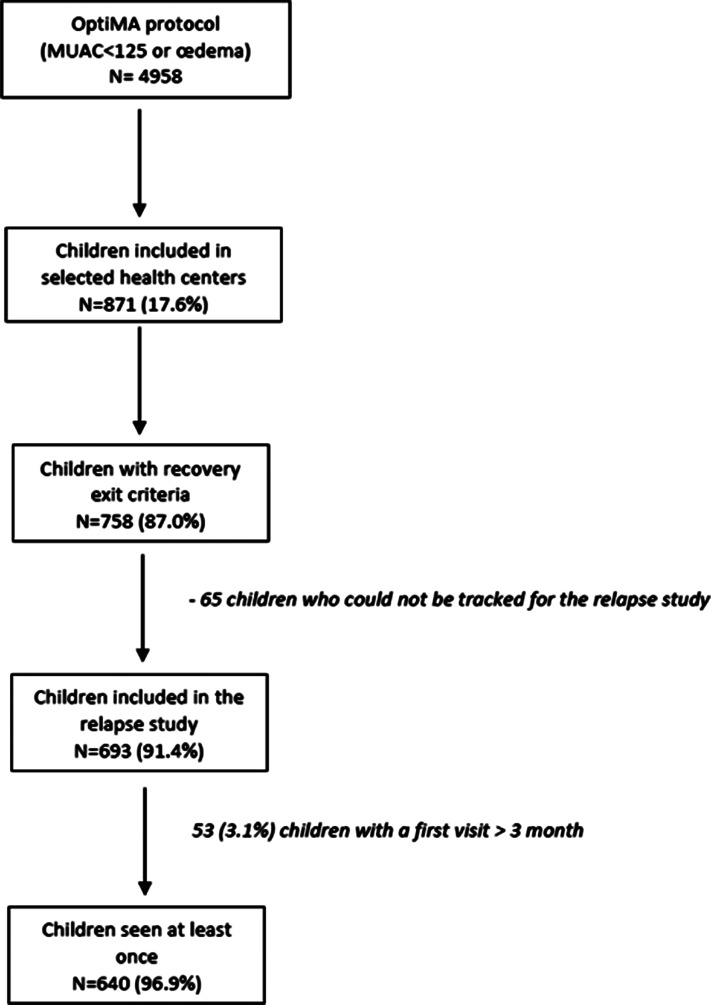
Flowchart of children recovering under the OptiMA protocol and included in the relapse study. Yako district, Burkina Faso, 2017–18.

About one-fifth of the children admitted during their first episode of AM under the OptiMA trial (*n* 4958) were included in the twelve selected health centres and eligible for the present study (Fig. 1). Of the 758 eligible children who recovered from the health facilities selected for this relapse study, 640 (84⋅4 %) were included in the analysis (Fig. 1). A total of 118 children (15⋅6 %) were excluded from the analysis, including 65 (8⋅6 %) who could not be tracked for participating in the relapse study and 53 (7⋅0 %) who had a first visit more than 3 months after their exit of the programme. These 53 children were all found to be alive, they were seen in median 5 months (IQR 4–6) after their exit and had a median MUAC of 129 mm (IQR 127–133 mm), and among them, only one child had a MUAC < 125 mm.

A comparison of maternal and child characteristics between children included in the relapse study (*n* 640), those eligible but not included (*n* 118) and all other children who recovered under the OptiMA protocol in the health facilities not selected for participating in the relapse study (*n* 2301), is presented in Table 1. These three populations were mostly comparable in terms of age, sex, maternal characteristics and child anthropometric data. However, there was a statistically significantly higher proportion of children living more than 10 km away from the health facilities among those eligible but not included (12⋅3 %) and those who recovered under the OptiMA protocol in the health facilities not selected (9⋅1 %) than among those actually followed in the relapse study (5⋅5 %).

**Table 1.** Comparison of maternal and child characteristics between children included in the relapse study (*n* 640), those eligible but not included (*n* 118) and those from health facilities not selected for participating in the relapse study (*n* 2301)

	Children included in the relapse study		Children eligible but not included in the relapse study		*P*-value^a^	Children from health facilities not included in the relapse study		*P*-value^b^
	*N* 640		*N* 118			*N* 2301		
	*n*	%	*n*	%		*n*	%	
Maternal characteristics at admission								
Maternal vital status (*n* 745)								
Alive	620	98⋅7	116	99⋅1	0⋅7	2232	98⋅8	0⋅9
Death	8	1⋅3	1	0⋅9		27	1⋅2	
*Missing data*	*12*	*–*	*1*	*–*		*42*	*–*	
Number of siblings								
0	2	0⋅3	0	0⋅0	0⋅8	7	0⋅3	0⋅4
[1–4]	326	54⋅2	64	55⋅2		923	57⋅5	
[4–12]	273	45⋅4	52	44⋅8		1260	42⋅1	
*Missing data*	*39*	*–*	*2*	*–*				
MUAC training								
Yes	455	72⋅6	86	74⋅8	0⋅6	1740	77⋅5	0⋅01
No	172	27⋅4	29	25⋅2		506	22⋅5	
*Missing data*	*13*	*–*	*3*	–		*55*	*–*	
Child characteristics								
Age (*month, mean (sd*))	15⋅2	(8⋅5)	15⋅9	(9⋅4)	0⋅4	15⋅0	(9⋅0)	0⋅5
Age category								
<24 m	511	79⋅8	92	78⋅0	0⋅6	1899	82⋅5	0⋅2
≥24 m	129	20⋅2	26	22⋅0		402	17⋅5	
Gender (*n* 757)								
Male	288	45⋅0	50	42⋅7	0⋅6	953	41⋅6	0⋅1
Female	352	55⋅0	67	57⋅3		1337		
*Missing data*			*1*	*–*		*11*	*–*	
Distance to the health centre (*n* 715)								
>10 km	568	94⋅5	100	87⋅7	<0⋅01	1997	90⋅9	<0⋅01
<10 km	33	5⋅5	14	12⋅3		200	9⋅1	
*Missing data*	*39*	*–*	*4*	*–*		*104*	*–*	
Child anthropometric characteristics								
MUAC at admission								
Mean (sd)	119⋅3	(4⋅1)	118⋅7	(5⋅2)	0⋅2	119⋅3	(4⋅7)	0⋅9
MUAC at admission category								
<115	81	12⋅7	21	17⋅8		272	11⋅8	0⋅7
115–119	139	21⋅7	22	18⋅6	0⋅3	480	20⋅9	
120–124	420	65⋅6	75	63⋅6		1549	67⋅3	
MUAC at discharge								
Mean (sd)	128⋅9	(3⋅7)	129⋅2	3⋅6	0⋅4	128⋅7	(3⋅4)	0⋅2
MUAC at discharge category								
125	76	11⋅9	10	8⋅5		271	11⋅8	
[126–128]	287	44⋅8	53	44⋅9	0⋅5	1049	45⋅6	0⋅9
≥129	277	43⋅3	55	46⋅6		981	42⋅6	

Yako district, Burkina Faso, 2017–18.

a Comparison of children included in the relapse study *v.* those eligible but not included.

b Comparison of children included in the relapse study *v.* those who recovered from health facilities not included in the relapse study.

The frequency of bi-weekly home visits was respected for the majority of children, as 66⋅1 % were seen at each planned visit and 80⋅2 % completed at least five of the six planned visits. At 3 months post-recovery, 545 children (85⋅2 %) were alive, 2 (0⋅3 %) were deceased and 93 (14⋅5 %) had not been seen at this last visit.

**Fig. 2.**
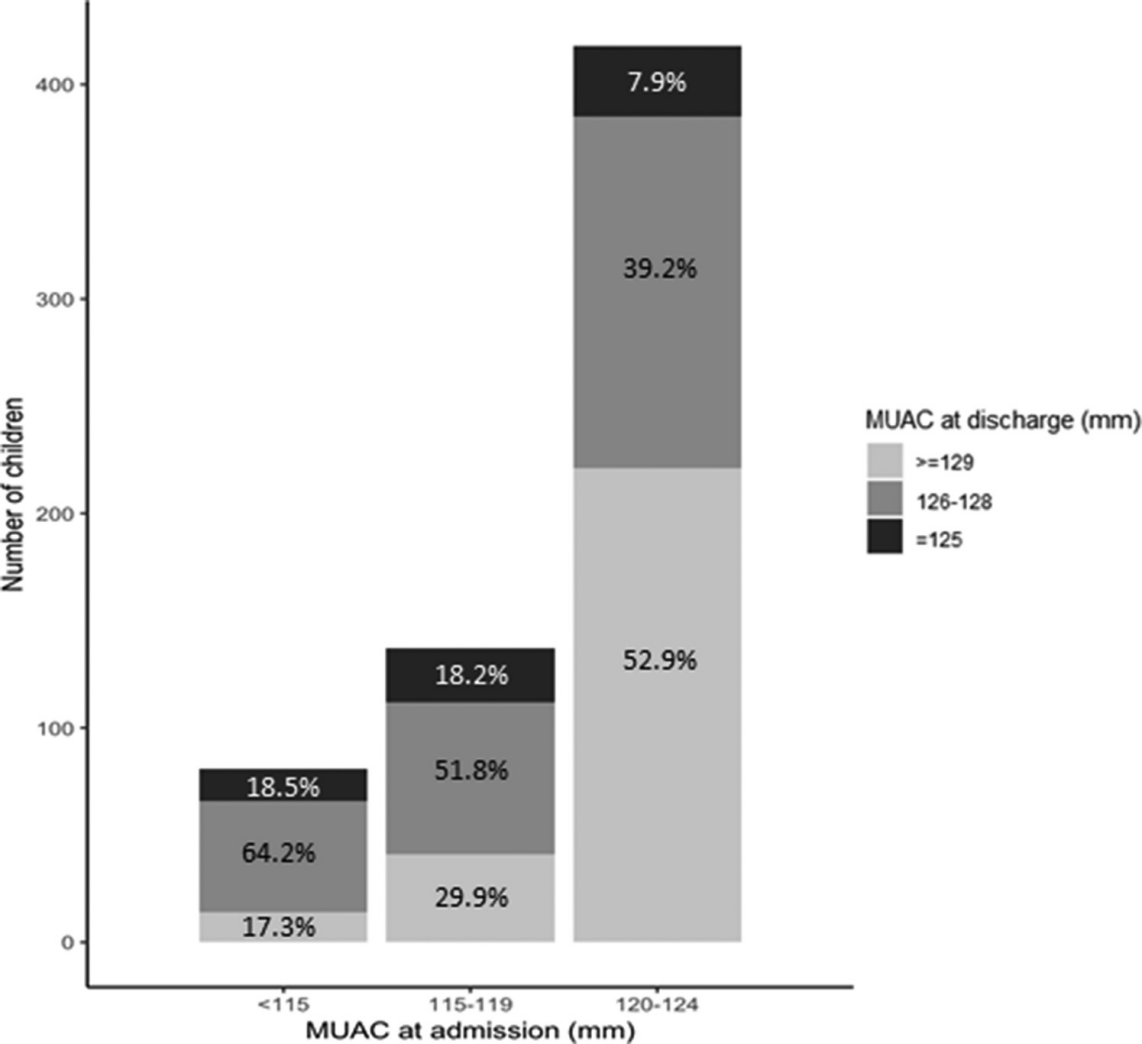
Proportion of children in MUAC categories at discharge according to MUAC categories at admission among children who recovered from the OptiMA strategy in the randomly selected health facilities (*n* 640). Yako district, Burkina Faso, 2017.

Fig. 2 shows the proportion of children in MUAC categories at discharge according to MUAC categories at admission among children who recovered from the OptiMA strategy in the randomly selected health facilities. Overall, 17⋅3, 29⋅9 and 52⋅9 % of children were discharged with a MUAC ≥ 129 mm among those who had been admitted with a MUAC < 115, 115–119 and 120–124 mm, respectively. The median MUAC at discharge was 127⋅1 mm (IQR 126–128 mm), 128⋅0 mm (IQR 126–129 mm) and 129⋅6 mm (IQR 127–132 mm) among children admitted with a MUAC < 115, 115–119 and 120–124 mm, respectively.

Table 2 shows the prevalence at 3 months, cumulative incidence and incidence rates of relapse per 100 child-months. A total of 44 (6⋅9 %) children relapsed with a MUAC < 125 mm in the 3-month following discharge, including 39 (6⋅1 %) who relapsed with a MUAC between 120 and 124 mm, 2 (0⋅3 %) who relapsed with a MUAC between 115 and 119 mm, 3 (0⋅5 %) who relapsed with MUAC < 115 mm. The relapse prevalence at 3 months post-recovery was 2⋅4 % (95 % CI 1⋅2, 3⋅5) among the 545 children seen at this last visit. The cumulative incidences of relapse at 1- and 3 months post-recovery were 1⋅9 % (95 % CI 1⋅0, 3⋅0) and 6⋅8 % (95 % CI 5⋅2, 8⋅8), respectively, among the 640 children follow-up in the present study. The median time to relapse was 42 d (IQR 28⋅0–59⋅5).

**Table 2.** Prevalence, cumulative incidence and incidence rates (per 100 child-months) according to MUAC at admission and at relapse among children who recovered from the OptiMA strategy in the randomly selected health facilities (*n* 640)

	*n*/*N*	% [95 % CI]
Prevalence		
Prevalence at 3 months	13/545	2⋅4 [1⋅2, 3⋅5]
Cumulative incidence		
Cumulative incidence at 1 month (*n* 640)	12/640	1⋅9 [1⋅0, 3⋅0]
Cumulative incidence at 3 months % (*n* 640)	44/640	6⋅8 [5⋅2, 8⋅8]
Incidence rates (per 100 child-months)		
MUAC at admission to MUAC at relapse^a^		
MUAC < 125mm to MUAC < 125 mm	44/1740	2⋅5 [1⋅9, 3⋅3]
MUAC < 125 mm to MUAC < 115 mm	3/1740	0⋅2 [0⋅1, 0⋅4]
MUAC < 125 mm to MUAC 115–124	41/1740	2⋅4 [1⋅7, 3⋅1]
MUAC < 115 mm to MUAC < 125 mm	8/213	3⋅7 [1⋅9, 6⋅8]
MUAC < 115 mm to MUAC 115–124	7/213	3⋅3 [1⋅6, 6⋅1]
MUAC < 115 mm to MUAC < 115 mm	1/213	0⋅5 [0⋅1, 1⋅7]
MUAC 115–124 mm to MUAC < 125 mm	36/1527	2⋅4 [1⋅7, 3⋅2]
MUAC 115–124 mm to MUAC 115–124 mm	34/1527	2⋅2 [1⋅6, 3⋅0]
MUAC 115–124 mm to MUAC < 115 mm	2/1527	0⋅1 [0⋅0, 0⋅4]

Yako district, Burkina Faso, 2017–18.

a MUAC at admission during the first admission in OptiMA programme to MUAC at relapse.

The number of person-time was 1740 (49*1 + 82*2 + 509*3), 213 (8*1 + 14*2 + 59*3) and 1527 (41*1 + 68*2 + 450*3) among children included with a MUAC < 125 mm, a MUAC < 115 mm and a MUAC between 115 and 124 mm in the OptiMA programme, respectively.

The global incidence rate of relapse among children included with a MUAC < 125 mm was 2⋅5 per 100 child-months (95 % CI 1⋅9, 3⋅3). The highest incidence rate of relapse was found among children admitted with a MUAC < 115 mm (3⋅7 per 100 child-months; 95 % CI 1⋅9, 6⋅8). The incidence rate of relapse among children admitted with a MUAC between 115 and 124 mm was 2⋅4 (95 % CI 1⋅7, 3⋅1) per 100 child-months.

The multivariable analysis showed that the incidence of relapse was positively associated with hospitalisation (Table 3), with an adjusted Hazard Ratio (aHR) of 2⋅06 (95 % CI 1⋅01, 4⋅26) for children who had an inpatient stay at any point during treatment compared with children who did not.

**Table 3.** Maternal and child characteristics associated with the 3-month incidence of relapse among children who recovered from the OptiMA strategy in the randomly selected health facilities (*n* 640)

	*n*/*N*	%	Univariate analysis		Multivariate analysis	
			Crude HR (95 % CI)	*P*-value	Adjusted HR (95 % CI)	*P*-value
Child socio-demographics characteristics						
Age category (*n* 638)						
<24 m	36/511	7⋅0	1	1⋅0		
≥24 m	8/129	6⋅2	1⋅00 [0⋅46, 2⋅17]			
Sex (*n* 638)							
Female	29/352	8⋅2	1	0⋅1	1	0⋅2
Male	15/288	5⋅2	0⋅63 [0⋅34, 1⋅19]		0⋅65 [0⋅35, 1⋅22]	
Distance from health centre (*n* 599)							
<10 km	39/568	6⋅9	1	0⋅5		
>10 km	4/33	12⋅1	0⋅68 [0⋅24, 1⋅96]			
Mother's characteristics at admission						
Mother's status (*n* 626)						
Death	0/8	0⋅0	–	–		
Alive	43/620	6⋅9				
MUAC training (*n* 626)						
Yes	35/455	7⋅7	1	0⋅6		
No	9/172	5⋅2	1⋅27 [0⋅58, 2⋅76]			
Number of siblings (*n* 599)						
≤2	16/220	7⋅3	1	0⋅7		
>2	24/381	6⋅3	0⋅88 [0⋅47, 1⋅66]			
Anthroprometric data						
MUAC at admission (mm) (*n* 638)						
120–124	21/420	5⋅0	1		1	
115–119	15/139	10⋅8	2⋅10 [1⋅07, 4⋅09]	0⋅04	1⋅85 [0⋅94 3⋅65]	0⋅2
<115	8/81	9⋅9	2⋅16 [0⋅95, 4⋅91]		1⋅69 [0⋅68, 3⋅85]	
Weight gain at discharge (kg) (*n* 628)						
≤2⋅5	17/217	7⋅8	1	0⋅6		
>2⋅5	27/413	6⋅5	0⋅85 [0⋅46, 1⋅57]			
Length of stay (week) (*n* 638)						
≤5⋅5	18/400	4⋅5	1	0⋅003		
>5⋅5	26/240	10⋅8	2⋅53 [1⋅38, 4⋅63]			
Hospitalisation						
Hospitalisation						
No	33/556	5⋅9	1	0⋅01	1	0⋅05
Yes	11/84	13⋅1	2⋅36 [1⋅19, 4⋅67]		2⋅06 [1⋅01, 4⋅26]	

Yako district, Burkina Faso, 2017–18.

## Discussion

To the best of our knowledge, this is the first cohort evaluating the incidence of relapse among 6–59 months malnourished children who had recovered from a nutrition programme solely based on a MUAC admissions criteria of <125 mm and with gradual reduction of RUTF dosage. We found an overall cumulative incidence of relapse of 6⋅8 % (95 % CI 5⋅2, 8⋅8) in the 3-month following recovery under the OptiMA strategy. Overall, the incidence rate of relapse was 2⋅5 (95 % CI 1⋅9, 3⋅3) per 100 child-months; for children admitted with a MUAC < 115 mm, it was 3⋅7 (95 % CI 1⋅9, 6⋅8) per 100 child-months and for children admitted with a MUAC between 115 and 124 mm, it was 2⋅4 (95 % CI 1⋅7, 3⋅2) per 100 child-months. The vast majority (88⋅6 %) of relapses were detected early with a MUAC between 120 and 124 mm, and very few (0⋅5 %) were MUAC < 115 mm. We also showed that having an inpatient stay at any point during treatment (aHR 2⋅06 95 % CI 1⋅01, 4⋅26) significantly increased the risk of relapse when compared with children who had not.

The incidence of relapse following recovery under the OptiMA protocol was relatively low (6⋅8 %; 95 % CI 5⋅2, 8⋅8). Systematic reviews have recently highlighted the lack of standard definition, as well as recommended indicators to measure or period for follow-up, which contributes to an inability to compare relapse rates across contexts^(16,17)^. For instance, incidence among children who recovered under the OptiMA strategy with a MUAC < 115 mm and who relapsed with a MUAC less than 115 mm was as low as 1⋅2 % (95 % CI 0⋅0, 3⋅3). This result is somewhat similar to the 1⋅9 % (95 % CI 0⋅4, 5⋅6) relapse rate found in a study in Malawi that defined relapse as MUAC ≤ 115 mm and used the same 3-month, bi-weekly follow-up of children at home^(4)^. Children were exposed to RUTF treatment for similar periods in these two studies: medians of 49 d (IQR 35–77) and 53⋅5 d (IQR 40⋅5–70⋅0) in Malawi and Burkina Faso, respectively. While a standard definition and acceptable threshold of relapse are needed, the low incidence of relapse observed in the present study suggests that the OptiMA strategy is promising.

Stobaugh *et al.* recommended twice monthly follow-up by CHWs for 3 months after discharge for identifying relapse occurrence^(16)^. More recently, the CORTASAM recommended at least monthly measurement for 6 months post-discharge by requesting caregivers to return to the location of the treatment programme or performed by CHWs in the community^(14)^. In the present study, the majority (88⋅6 %) of relapses were detected early with a MUAC between 120 and 124 mm. This early detection and the low incidence of severe episodes of relapse could be explained by the home-based routine follow-up in place and by the expanded definition of relapse with a MUAC criterion <125 mm. Systematically using CHWs for identifying such relapse episodes may be cost-prohibitive and may not be feasible at a scale, however. Asking mothers to return to the health facility with their child does not seem relevant in this context where households are not necessarily close to these health facilities. Given that training caretakers to use MUAC bracelets to screen their own children for acute malnutrition led to earlier treatment admission compared with screenings performed by CHWs^(18)^, we believe that caretakers should be re-trained at discharge to screen their children every 2 weeks or 1 ×  per month from home with MUAC bracelets so that relapse cases could be detected early.

The incidence rate of relapse estimated in the present study might have been underestimated by concurrent programming for MAM. The World Food Program sporadically distributed ready-to-use supplemental food in several health facilities during the study period, targeting children with a MUAC >125 mm and a WHZ < −2 at the exit from the OptiMA protocol. It was not possible to determine which children participated in this supplementation, which may have contributed to a lower relapse rate.

The number of children with incomplete follow-up in the present study was high (*n* 118, 15⋅6 %), but 53 (45 %) were found to be alive beyond 3 months post-recovery. Children lost to follow-up were similar to children who were followed in terms of age, sex and anthropometric measures. However, children living 10 km or more from health facilities were more likely to be lost to follow-up than children who lived <10 km from health facilities (12⋅3 and 5⋅5 %, respectively). These children living far from a health centre in isolated rural villages are probably at a higher risk of food insecurity, and this non-representativeness of those lost to follow-up may also be contributed to an underestimation of the overall relapse rate.

To our knowledge, this is the first time that an increased risk of relapse among children hospitalised at least once was shown. The MUAC category at admission was not statistically associated with relapse in the final multivariable model (*P* 0⋅2), which was probably due to a lack of statistical power. Having lower anthropometric measures at admission or discharge is the most common risk factor associated with relapse^(4,5,7)^ and a higher MUAC at discharge increases favourable outcomes^(19)^. Children with lower MUAC at admission have longer durations of treatment and higher proportional weight gains than children admitted with higher MUAC^(4)^, but these children admitted with a lower MUAC also have a lower MUAC at discharge. In the present study, the median MUAC at discharge was 127⋅1, 128⋅0 and 129⋅6 mm among children admitted with a MUAC <115, 115–119 and 120–124 mm, respectively. This highlights the issue of the unique threshold at 125 mm used for admission, recovery and relapse under the OptiMA strategy, which necessarily increases the risk of relapse among children admitted with a low MUAC. However, imposing higher MUAC discharge criteria would increase both lengths of stay and the proportion of children classified as non-responders. A possibly more efficient course of action would consist of training caretakers to detect relapse early, with a special focus on caretakers whose child was admitted for treatment with MUAC <115 mm or who had an inpatient stay at any point during treatment.

## Conclusion

The incidence of relapse following recovery under the OptiMA protocol was relatively low, suggesting that the OptiMA strategy is promising, but the lack of a standard relapse definition does not allow for a comparison with other studies. Relapse should be a more widely used outcome to assess malnutrition treatment programme effectively. Home visits by CHWs during 3 or 6 months can detect relapse at an early stage, but re-training caretakers at discharge to screen their children for relapse by MUAC at home could potentially be more effective and less costly. Closer follow-up with caretakers whose children admitted with MUAC <115 mm or had an inpatient stay at any point during treatment could be an efficient course of action. Further work is still needed to better define risk groups and factors associated with relapse.
